# Epidemiology of the last Wednesday Eve Festival injuries in East Azerbaijan Province, Iran

**Published:** 2019-08

**Authors:** Saeid Pour Doulati, Jabraeil Sharbafi, Mohsen Pasha, Fatemeh Anvari, Mitra Yeganeh, Maliheh Talebi

**Affiliations:** ^*a*^ Center for Non-Communicable Diseases Control and Prevention, East Azerbaijan Province Health Center, Tabriz, Iran.; ^*b*^ Alzahra Hospital, Tabriz Medical Sciences University, Tabriz, Iran.

## Abstract

**Background:**

Iranian people have been celebrating the last Wednesday Eve Festival (Chaharshanbeh Soori) from Zoroastrian tradition (1725 BC). This ceremony, which had previously comprised a wide range of socially pleasant and well-accepted traditions, has now been changed into a scene of horror and danger by fireworks that threaten the physical, mental and social health of the community. Purpose: The purpose of this study was to investigate the epidemiological aspects of injuries due to Chaharshanbeh Soori in East Azerbaijan Province, Iran.

**Methods:**

This is a descriptive epidemiological study. Data on injuries caused by Chaharshanbeh Soori events were collected by using a national questionnaire in the emergency words of all public and private hospitals from March 15 to April 15, 2018. In this study, demographic and epidemiological variables including age, sex, education, the position of the injured person, living area, injured organ, the type of injury, and outcome of the injury were collected. Data were analyzed using Excel 2013 software for descriptive statistics.

**Results:**

A total of 318 injured people have been registered (incidence rate of 9 per 100,000 population). 80% of them were male and 20% were female. 53.8% of the injured people were under 20 years of age. 90% of the injured lived in urban areas, 7% in rural areas and 3% in suburban areas. 65% of injuries occurred during the use of firecrackers, 32% occurred for whom watching or passing, and 3% occurred during the construction of firecracker. 62% were uneducated or under diploma, 29% had diplomas or associate degree, and 9% had a bachelor or master degree. In terms of the type of injury, 57% suffered burns, and 43% had cuts or wounds. In total 289 outpatients were treated as outpatients, 28 were hospitalized as inpatients, one led to disability and death was not reported.

**Conclusions:**

Considering that most injuries are made by urban boys under the age of 20 who use the firecrackers as a means of happiness, effective interventional planning for them and their parents is necessary by health and safety officials of the community using the (successful and unsuccessful) past experiences.

**Keywords:**

Wednesday Eve Festival, Persian, Firework injury

